# Changing poor mothers' care-seeking behaviors in response to childhood illness: findings from a cross-sectional study in Granada, Nicaragua

**DOI:** 10.1186/1472-698X-10-10

**Published:** 2010-06-01

**Authors:** Kayako Sakisaka, Masamine Jimba, Kyo Hanada

**Affiliations:** 1 Takemi Program in International Health, Department of Global Health and Population, Harvard School of Public Health, Boston, Massachusetts USA; 2 Department of Community and Global Health, Graduate School of Medicine, The University of Tokyo, Tokyo, Japan; 3 Human Development Department, Japan International Cooperation Agency (JICA), Tokyo, Japan

## Abstract

**Background:**

In 2008, approximately 8.8 million children under 5 years of age died worldwide. Most of these deaths occurred in developing countries, but little is known about poor mothers' care-seeking behaviors for their children.

We examined poor mothers' care-seeking behaviors in response to childhood illness, and identified factors affecting their choices. We also assessed mothers' perception of the medical services and their confidence in the health care available for their children.

**Methods:**

We carried out a community-based cross-sectional study with structured questionnaires. Participants were 756 mothers and their young children (0-23 months) in Nandaime municipality, Granada province, Nicaragua. We took the children's anthropometric measurements and we assessed the mothers according to their income. We divided them into 3 global absolute poverty categories (income: <1 USD/day, 1-2 USD/day, >2 USD/day), and 4 quintile.

**Results:**

When a child showed symptoms of illness, most mothers (>75%) selected public health facilities as their first choice. More than half (>58%) were satisfied with the medical services, but the poorest mothers expressed more dissatisfaction (p = 0.003), when we divided the participants into 4 quintiles groups according to their income. In the poorest group, the main reasons for dissatisfaction were cost (46.6%), and distance to the facilities (25.8%). Almost half (41.3%) of mothers lacked confidence in the health care offered to their child, while most of the wealthiest mothers (75.7%) did have confidence in it (p = 0.001). The poorest mothers showed greater interest in health education than the wealthiest (86.2% vs. 77.8%) (p = 0.015). We found that poor mothers (≤2 USD/day) changed their second choice for care in a positive direction. Factors affecting the change in second choice were the child having symptoms of respiratory disease (AOR, 2.51; 95% CI, 1.28-4.90, p = 0.007), visiting health post as the first choice (AOR, 2.11; 95% CI, 1.26-3.53, p = 0.005), and experiencing a child death in the past (AOR, 2.05; 95% CI, 1.15-3.68, p = 0.016). Child stunting, mother's level of education, and past participation in health education programs did not affect.

**Conclusions:**

Determination of the severity of a childhood disease is a difficult task for mothers. The national rural health system was functioning, yet the services were often limited. We should consider the feasibility of providing a more effective primary care system for the poor.

To encourage mothers' care-seeking behaviors in poor settings, the referral system and the social safety net need to be strengthened. Poor mothers need further education about the danger signs of childhood illness.

## Background

In 2008, approximately 8.8 million children under 5 years of age died worldwide. [1]. Most of these deaths occurred in developing countries [2,3]. An estimated 2.7 billion people are still living on less than 2 US dollars a day [4]. The major causes of child mortality are pneumonia, diarrhea, malaria, and other infectious diseases [3,5]. Deaths from these diseases might be preventable if early and appropriate treatment were rendered [6]. Mothers in developing countries, however, often do not have sufficient knowledge of signs that their child's health is in danger, or of appropriate treatments, or access to appropriate health services [6]. Poor mothers are also more likely to live in remote areas, which can lead to delays in seeking care, and to fatalities. A mother's care-seeking behavior is therefore particularly important in resource-poor countries.

Kroeger [7] provided a holistic framework for examining care-seeking behaviors, which is applicable for developing countries. His framework covers major dimensions of health-seeking behaviors, namely, sociodemographics, physical accessibility, medical cost for treatment, women's autonomy, and given health service factors.

Existing studies have shown that factors associated with a mother's care-seeking behaviors when faced with childhood illness are as follows: the mother's level of education [8-10], economic status [8,11,12], mother's age [13] ethnicity [13-17], distance to the health care facility [9,18,19], the child's age [20], birth order of the child [21,22], child nutritional status [12,23], lack of recognition of the severity of the illness [13,22,24], the presence of diarrhea [25] or respiratory disease [14,24], prior participation in health education [8,26], knowing a medical doctor [27], and the quality of the health care services provided [28,29].

Nicaragua is one of the poorest countries in Latin America and the Caribbean, followed by Haiti [4]. Its per capita gross national income (GNI) was 980 US dollars (USD) (2007) [4], which is one-third the regional average [4]. Moreover, Nicaragua has huge economic inequalities [4]. The Gini coefficient in Nicaragua was reported to be 0.54 in 2006 (0 = perfect equality, 1 = perfect inequality), with the wealthiest 20% earning approximately 60% of the country's total income [4]. Approximately 50-60% live below the extreme poverty line (living on less than 1.25 US dollars per day) [30].

Nevertheless, between 1980 and 2007, Nicaragua has succeeded in reducing its infant mortality rate (IMR) by almost two-thirds, from 82/1,000 to 28/1,000 and the under-5 years of age child mortality rate (U5MR) from 113/1,000 to 35/1000[2]. However, huge domestic disparities remained. The IMR in the richest quintile was 16/1,000, while that of the poorest quintile was 50/1,000 [4]. Similarly, the U5MR was 19/1,000 in the richest quintile and 64/1,000 in the poorest quintile [4]. The majority of poor mothers live in remote rural areas where medical services are scarce, and little is known about how they behave in seeking medical care when their children get sick.

We examined poor mothers' care-seeking behaviors in response to childhood illness, and identified factors affecting their choices. We also assessed mothers' perceptions of the medical services and their confidence in the health care offered to their children.

## Methods

### Study design

We carried out a cross-sectional study using face-to-face interviews with a structured questionnaire for mothers. In addition, we conducted anthropometric measurements of the target children.

### Study site

We selected Nandaime municipality in Granada province as our study site. Granada is 1 of the 17 provinces in Nicaragua. This study was conducted as the baseline survey of the Project for Strengthening Community Health in Granada province, supported by Japan International Cooperation Agency (JICA) [31]. In addition, the proportion of child stunting in Granada was similar to the national average [32]. For these reasons, we selected this area.

Granada province is located approximately 70 km southeast of the capital city Managua. In the year 2002, the total population of Granada province was about 170,000 [33]. Granada province consists of 4 municipalities: Granada, Nandaime, Diriomo and Diriá. We selected Nandaime municipality for its larger population size, the accessibility of its households, and its residents' ability to communicate in Spanish. The estimated population in Nandaime was nearly 37,500 [33]. The total number of households was about 7,400 [33]. Nandaime municipality had about 50 communities in total [33].

### Target population

We targeted mothers and one of their children under 2 years of age (0-23 months). Inclusion criteria for our study population were mothers and children aged 0 to 23 months living in the target area, and who were at home during the household visit. Since child nutritional status is more likely to deteriorate between aged 12 to 18 months, this is a crucial period for child mortality [34]. Exclusion criteria were caregivers who were only casually taking care of the children, and those who did not agree to provide informed consent.

Since we intended to include mothers who did not use health centers as well as those who did, we planned house-to-house visits for this study. We sought to access the more vulnerable, and poor mothers who cannot read or write.

### Sampling

#### Sample size calculation

We calculated the required sample size from the existing proportion of child stunting (35%) in rural provinces in Nicaragua [35]. The proportion of moderate stunting in children under 5 years old in Granada was about 25%, underweight was 12%, and wasting was 2% [32].

We adopted the formula provided by the guidelines of the World Health Organization (WHO) [36]. The formula was as follows:

n > z^2 ^× (p) × (1-p)/c^2^

z = z value (1.96 for 95% confidence level)

p = percentage picking a choice, we set at 35% (existing child stunting level) = 0.35

c = confidence interval.

We substituted the prevalence of child malnutrition at 35% in the formula and set <0.05 as the significance level. The required minimum sample size was 350. We planned to observe the disparities between urban and rural areas, and our minimum sample size was doubled to 700 (350 × 2).

### 2-stage cluster sampling

We used systematic random sampling. Since we planned to conduct community-based, household visit survey, we adopted 2-stage cluster sampling [37]. We checked the official number of children aged 2 years old in the municipal health office data. To ensure the minimum 700 samples, we needed to include many communities.

The urban area of Nandaime municipality is located at the northern part of the study area. In addition, we had to recruit rural mothers and children from the eastern, southern, and western parts of the target area. Due to geographical inaccessibility, several clusters were excluded. We visited all of the households in the selected areas, asking whether mothers and children of eligible age were there. If the necessary number of children was not obtained, we added the nearest communities in the target areas. In total, 37 study clusters were selected, comprising all 10 urban communities and 27 rural communities out of 51 communities [37].

### Questionnaire survey

The questionnaire was prepared based on the Demographic and Heath Survey, and an existing child health questionnaire survey used in rural Nicaragua [38]. After pre-testing in the field, certain modifications were made by the staff of Naidaime municipal health officers. The questionnaire consisted of the following parts: anthropometric data of the child, socioeconomic and demographic data of the respondent (mother), maternal and child health, health-seeking behaviors of the mother, monthly medical expenditures, utilization of health facilities, satisfaction with given health services, and experiences with willingness to attend health education programs.

### Definition of poverty in this study

We used two poverty indices in this study: absolute poverty and relative poverty. For the absolute poverty index, we defined 3 poverty groups based on the World Bank's categories [3]. Group 1: < 1 US dollar (USD) per day (extreme poverty level, monthly household income < 30 USD); Group 2: 1 to 2 USD per day (monthly household income, 30-60 USD); and Group 3: > 2 USD per day (monthly household income > 60 USD). We defined people living on less than 1 USD per day as 'the poorest,' and the 1-2 USD per day group as 'the poor,' and those living on more than 2 USD per day as the 'not poor.' Since the Economic Commission for Latin America and the Caribbean (ECLAC) defines the poverty line as living on less than 2 USD per day, we included those who are living on less than 2 USD per day in the poor. For the relative poverty index, we divided the respondents into quartile groups by monthly income level.

### Definition of symptoms of diseases

We adopted the definitions common to WHO and the United Nations International Children's Fund (UNICEF) for symptoms of diarrhea, respiratory disease, and fever. The definition of diarrhea was watery stools that occurred more than 3 times during any 24-hour period [39]. Symptoms of respiratory disease were defined as difficulty in breathing and continuous cough [32]. Fever was defined as having a temperature higher than 38°C. A recall period was adopted of 14 days (2 weeks) prior to the study, based on the DHS questionnaire [32].

### Definition of positive change of care-seeking behavior

We defined positive care-seeking behavioral change as changing from doing nothing, self-medication, or home remedies to consulting health personnel, visiting health facilities where health professionals were assigned, and changing from using a rural health post to health center or a private clinic.

### Health center and health posts

In this area, there was 1 public health center staffed by physicians. Rural health posts were staffed only by paramedics. There were several health posts and private clinics in the study area.

### Anthropometric measurement

Research teams measured the children's weight and height (length). Child weight was measured to the nearest 0.1 kg using a digital scale, TANITA THD652 model (in 100-g increments) [40]. Child height (length) was measured using tape measures and wooden boards (width 25.3 cm, length 36.8 cm, thickness 5 mm). For those children who could not stand alone, the other team member and the mothers assisted in extending the child by bending the ankles on the dining table (recumbent position), or appropriate tables in the house. Children who could stand alone were measured standing. Researchers measured the children's height to the nearest 0.1 cm.

### Evaluation of child nutritional status: z-scores

We used the National Center for Health Statistics (NCHS)/WHO reference data [34,36], which are commonly used to evaluate target child nutritional status. Since NCHS/WHO reference data were collected from many countries including developing countries, the data show a very high consistency in the standard deviation. Even under conditions of extreme famine, the value of the standard deviation of the z-score is very close to unity [34].

The height-for-age z-scores (HAZ) below minus 2 SD (<-2 HAZ) represent stunting, indicating chronic malnutrition. The weight-for-age z-scores (WAZ) below minus 2SD (<-2 WAZ) represent acute malnutrition [34]. The weight-for-height z-scores (WHZ) below minus 2SD (<-2 WHZ) are categorized as wasting, which indicates a recent and severe process of weight loss, often associated with acute starvation or severe disease [34].

### Data collection

We conducted the field survey from February to March 2002. We assigned 2 researchers to each team. At least 1 researcher was a medical doctor or registered nurse who was familiar with anthropometric measurements of infants. Every day, 8 teams visited households to conduct interviews and take anthropometric measurements. Prior to the field research, we carried out 2 days of training for the 16 assistant researchers. Since all assistant researchers worked or lived in the study area, they had sufficient knowledge about the social and health situation in the study site. We had a meeting every morning before starting data collection, and each afternoon after the daily work. We checked and confirmed the data sheets every day, and shared important information during staff meetings.

### Data management

The principal researcher (KS) and a core staff of research assistants checked the collected data sheets and entered the data. Data quality check and data cleaning were also performed.

### Data analysis

We calculated frequencies, proportions, means, standard deviations for categorical variables, and medians accordingly for continuous variables. To explore associations between two variables, we used a chi-square test for categorical variables. To compare 3 continuous variables we employed analysis of variance (ANOVA). Then, we calculated crude odds ratios (COR) with 95% confidence intervals (CI) using bivariate logistic regression analysis. To determine the predictors, we employed multiple logistic regression analysis. We calculated adjusted odds ratios (AOR) and 95% CI for the final model. We used PASW Statistics 18.0 for Windows [41], and EPI Info.3.5.1 for calculating child z-scores.

### Ethical considerations

We obtained informed consent verbally from all mothers who participated in the interviews. The Granada provincial health office (SILAIS Granada), Ministry of Health, Nicaragua, reviewed and approved the study protocol. On the Japanese side, the advisory committee for the Project for Strengthening Community Health in Granada, Nicaragua, (PROGRA) approved the study protocol.

## Results

### Sociodemographics and child health-related information of the respondents

The total number of participating households was 756 (response rate, 100.0%; total covered population, n = 5,198). Table 1 shows the sociodemographic and child health-related information of the respondents.

**Table 1:** Sociodemographics, and child health-related information of the respondents

Variables		Number	%
**Age of mothers(n = 756)**		(Mean) 24.3 ± 5.7 yrs.	
<20		165	21.8
20-29		438	57.9
30-39		138	18.3
40-49		15	2.0
**Number of child**		(Mean) 2.5 ± 1.9	
**Age of child (n = 756)**		(Mean) 12.1 ± 6.7 months	
0-5 months		169	22.4
6-11 months		173	22.9
12-18 months		270	35.7
19-23 months		144	19.0
**Gender of child (n = 756)**			
boy		383	50.9
girl		370	49.1
**Birth order of the child (participants) (n = 756)**			
1-2		468	61.9
3+		288	38.1
**Years of school attendance of mothers(n = 756)**		(Mean) 5.8 ± 3.6 yrs.	
no formal education		76	10.1
primary school (1-6 yrs)		264	34.9
secondary school (7-12 yrs)		311	41.1
higher education (≥13 yrs)		105	13.9
**Mother's occupation (n = 756)**			
housewife		675	89.3
**Number of family per household**		(Mean) 6.8 ± 3.3	
**Average monthly income (in USD)(n = 744) **(Median)800 (= 58.0 USD) [IQR:500-1300]			
<500 (<36.3 USD)		137	18.5
500-799(36.3-58.0 USD)		170	22.8
800-1299(58.0-94.3 USD)		248	33.3
≥1300(≥94.3 USD)		189	25.4
**Distance to the nearest health facilities (n = 756)**			
<30 minutes on foot		379	50.1
≥30 minutes on foot		219	29.0
Not walking distance (by vehicle, motorbike etc.)		158	20.9
**Child stunting (<-2HAZ)(n = 754)**			
<-2HAZ		227	30.1
≥-2HAZ		527	69.9
**Child underweight (<-WAZ)(n = 755)**			
<-2WAZ		78	10.3
≥-2WAZ		677	89.7
**Child wasting (<-WHZ)(n = 754)**			
<-2WHZ		37	5.0
≥-2WHZ		717	95.0
**Had symptoms of diarrhea (target child)^b^**	yes	129	17.1
**Had symptoms of respiratory disease (target child)^b^**	yes	524	69.3
**Had experience of child death before**	yes	128	16.9
**Participated in health education before**	yes	286	37.8
**Proportion of medical cost to monthly income **(Median) 10.0% [IQR:4.2-23.7]			

^a^1 USD = 13.77 Cordobas (2002)

^b ^within the last 14 days prior to this study

The mean age of the mothers was 24.3 (±5.7) years old, the mean number of children was 2.5 (±1.9), and the mean age of the target child was 12.1 (±6.7) months. More than half (61.9%) of the target children were the first or second child in the family. The mean duration of the mothers' school education was 5.8 (±3.6) years, and 10.1% of the mothers had no school education. A majority were housewives (89.3%). The median monthly household income was 800 Cordobas, which is equivalent to 58.0 USD (in 2002, 1 USD = 13.77 Cordobas, interquartile range [IQR]: 500-1300). This value was based on the verbal answers from mothers. The mean number of family members was 6.8 (±3.3). We selected almost the same sample size (minimum size: 328) from urban and rural areas in this study site, and half of the mothers (50.1%) answered that the nearest health facility was within 30 minutes' walking distance.

As for the children's nutritional status, 30.1% of the children were stunted, 10.3% were underweight, and 5.0% were wasting. Of the total children, 17.1% had symptoms of diarrhea and 69.3% had symptoms of respiratory disease within the last 14 days prior to the study. More than one-third of mothers (37.8%) had participated in a health education session in the past; however, 16.9% of mothers mentioned they had at least 1 child death experience before. Their median monthly medical cost rate was 10% (IQR: 4.2-23.7%).

### Differences among selected sociodemographics and health indicators by income level

Table 2 outlines factors associated with sociodemographics and health-related indicators by 3 income levels. The mean monthly income of Group 3 was approximately 5 times that of Group 1 (117.4 USD vs. 23.4 USD; p < 0.001). Maternal education was significantly higher in the more affluent groups (p < 0.001). The mean height for age z-score (HAZ) was -1.51 (SD, 1.65) in Group 1, -1.42 (SD, 1.58) in Group 2, and -0.69 (SD, 1.58) in Group 3 (p < 0.001).

**Table 2:** Differences among selected sociodemographics and health indicators by income level (n = 744)^1,2^

	Group 1	Group 2	Group 3	
	**<1 USD/day**	**1-2 USD/day**	**>2 USD/day**	
	**(n = 140)**	**(n = 252)**	**(n = 352)**	
**Studied variables**	**Mean (SD)**	**Mean (SD)**	**Mean (SD)**	***p*****-value**

Mean monthly household income (USD)	23.4 (6.6)	47.0 (8.8)	117.4 (72.5)	<0.001^a^
Mother's education (yrs)	4.1 (3.1)	4.8 (3.0)	7.1 (3.8)	<0.001^a^
Child height for age z-scores (HAZ)	-1.51 (1.65)	-1.42 (1.58)	-0.09 (1.58)	<0.001^a^
number and % <-2HAZ	52 (37.7%)	89 (34.5%)	86 (24.0%)	<0.001^b^
Distance to the nearest health facility	%	%	%	
<30 minutes on foot	42.9	39.5	60.6	<0.001^b^
≥ 30 minutes on foot	29.3	36	23.7	
Not walking distance	27.8	24.5	15.7	

^1^12 mothers did not answer about monthly income.

^2^We examined all other socio-demographics and child health status showed in Table 1.

Except above variables, others did not show any significant associations among 3 income groups.

^a^ANOVA (analysis of variance)

^b^Chi-square test

Regarding distance to the nearest health facility, more than half (60.6%) of Group 3 had within 30 minutes' walking distance, whereas more than half of the other 2 groups had to walk more than 30 minutes to the nearest health facilities (p < 0.001).

### **Mothers' perception of medical services, confidence in children's health care, and willingness to participate in health education by income quartile groups**

More than half of the mothers (>58%) were satisfied with the medical services available in the study area (Table 3). Affluent mothers were more satisfied than the poor. Poor mothers expressed more problems with medical services. In the poorest group, the money they had to pay (46.6%) and the distance to the medical facilities (25.8%) were the reasons for their dissatisfaction with medical services. On the contrary, the wealthiest group was more likely to express their dissatisfaction with their claims of long waiting times in health facilities (44.1%) and the standards of the health service they experienced (25.4%). Significant differences were found in the 4 groups (p = 0.003). In the poorest group, almost half (41.3%) of mothers lacked confidence in their child's health care, while a majority of (75.7%) the wealthiest mothers had confidence in their child's health care. Significant differences were found among the 4 groups (p = 0.001)

**Table 3:** Mothers' perception of medical services, confidence in child health care, willingness to participate in health education by income quartile groups (N = 744)^1^

Monthly income	1st quartile	2nd quartile	3rd quartile	4th quartile	
(Cordobas)	**<500**	**500-799**	**800-1299**	**>1300**	
(USD)	**(<36 USD)**	**(36-58 USD)**	**(59-94 USD)**	**(>94 USD)**	
	**(n = 137)**	**(n = 170)**	**(n = 248)**	**(n = 189)**	**χ^2^**
	**%**	**%**	**%**	**%**	**p-value**

**Satisfied**	58.0	58.2	64.5	69.3	0.083

**Dissatisfied**					
long waiting time	20.7	21.1	26.4	44.1	0.003**
money to pay	46.6	49.3	36.8	25.4	
distance	25.8	18.3	21.9	5.1	
quality of service	6.9	11.3	14.9	25.4	
**Confidence in child health care**					
yes	58.7	60.6	70.9	75.7	0.001**
no+ not so much	41.3	39.4	29.1	24.3	
**Willingness to participate in health education**					
yes	86.2	87.6	87.9	77.8	0.015*
no +not so much	13.8	12.4	12.1	22.2	

^1^12 mothers did not answer about monthly income.

*p < 0.05, **p < 0.01

As for the mothers' willingness to participate in health education, many of the mothers (>77%) were interested in health education. Over all, the poorest mothers showed a greater interest in health education than did the wealthiest (86.2% vs. 77.8%) (p = 0.015).

### Poverty and mothers' care-seeking behavior and childhood illness

Table 4 depicts the associations between the mothers' care-seeking behavior and child hood illness and economic status. The majority of mothers (> 75%) in all groups selected public health facilities (health center or health post) as their first choice. Wealthier mothers (Group 3) were more likely to use private facilities as their first choice (13.1%, p = 0.038). Regarding the second choice, more than half of the mothers (> 60%) would still visit public health facilities. Of the poorest mothers, however, 11.6% selected private clinics as their second choice. The proportion that would use self-medication and home remedies decreased as a second choice among all income groups.

**Table 4:** Mothers' care-seeking behaviors in response to childhood illness by 3 income levels

	Group 1	Group 2	Group 3	
Income	<1 USD/day	1-2 USD/day	>2 USD/day	χ^2 ^ p-value
	%	%	%	
**First choice**	**(n = 133)^1^**	**(n = 251)^2^**	**(n = 343)^3^**	
Public health facilities^4^	82.0	78.9	75.2	0.038*^a^
Self-medication, home care^5^	13.5	13.1	11.7	
Private clinic	4.5	8.0	13.1	
**Second choice**				
[first choice only]	12.3	15.1	18.2	0.157^a^
Public health facilities^4^	65.2	65.9	62.1	
Self-medication, home care^5^	10.9	8.7	5.4	
Private clinic	11.6	10.3	14.3	

^1^no illness = 7. ^2^no illness = 7. ^3^no illness7 + 15missing data..^4^Public health facilities: public health center + health post. ^5^Homecare: taking care in the house.

^a^Chi-square test

*p < 0.05

### Factors affecting change of second choice of care-seeking behavior among poor (≤ 2 USD/day) mothers

As shown in Table 5, we found several possible factors which may affect a change in the second choice of poor mothers seeking health care for their children in a positive direction: symptoms of respiratory disease (COR, 2.77; 95% CI, 1.43-5.35, p = 0.003), visiting health post first (COR, 2.24; 95% CI, 1.35-3.70, p = 0.002), and experiencing a child's death in the past (COR, 2.07; 95% CI, 1.17-3.64, p = 0.012). Child stunting, mother's level of education, and participation in health education in the past did not affect change in the mother's behavior. After adjusting for possible confounding factors, we identified the following 3 predictors: symptoms of respiratory disease (AOR, 2.51; 95% CI, 1.28-4.90, p = 0.007), visiting health post as the first choice (AOR, 2.11; 95% CI, 1.26-3.53, p = 0.005), and experiencing a child's death in the past (AOR, 2.05; 95% CI, 1.15-3.68, p = 0.016).

**Table 5:** Factors affecting the change in second choice^1 ^of poor (≤ 2 USD/day) mothers' care-seeking behavior (n = 392)

Variables	Crude Odds Ratio	95%CI	*p*-value	Adjusted Odds Ratio^2^	95%CI	p-value
Had symptoms of respiratory disease	2.77	[1.43-5.35]	0.003**	2.51	[1.28-4.90]	0.007**
Visiting health post first	2.24	[1.35-3.70]	0.002**	2.11	[1.26-3.53]	0.005**
Experiencing child death in the past	2.07	[1.17-3.64]	0.012*	2.05	[1.15-3.68]	0.016*
Participation in health education in the past	1.22	[0.71-1.94]	0.532			
Child stunting	1.17	[0.74-2.02]	0.436			
Living in rural area	1.06	[0.64-1.75]	0.824			
Mother's years of school education	0.81	[0.56-2.11]	0.809			

^1^Changed care-seeking behavior: from self-medication or home care to visit a health facility, from a health post to a public health center or private clinic, or from a public health center to a private clinic as a second care-seeking behavior.

^2^Multiple logistic regression. Adjusted for having symptoms of respiratory disease, visiting health post first, and experiencing child death in the past.

*p < 0.05, **p < 0.01

## Discussion

### Care-seeking behavior of poor mothers

We found that a majority of the target mothers utilize public health facilities first when they face a childhood illness (>75%). This surprisingly high rate of utilization might be attributable to the low consultation cost of public health service, and partly attributable to the regular community visits by government health staff members at least once a year during immunization campaign weeks, the '*Jornada.' *Not only do they immunize children, but they also give health advice to the mothers. Thus, close relationships had already been established between mothers and the medical staff in the area. Recent studies have demonstrated that home visits by the health workers may contribute to reduce childhood deaths in developing countries [42,43].

Our results were consistent with some previous studies showing popular utilization of government health services among poor people [44,45]. Contrary to the high utilization of public health services in this study area (Table 4), studies in Pakistan [20], Guatemala [46], and Vietnam [47] showed a lack of confidence in public health services by the people. The reasons were mainly a lack of health staff, low quality of service, and a shortage of drugs.

### Predictors of poor mothers' behavioral change

Of the 3 factors that were found to affect their behavior, the symptoms of respiratory disease might be regarded as a distressing sign for mothers [24,48]. Although we did not identify in detail the severity of the symptoms, mothers might know that respiratory disease is one of the common killers of younger children.

Choosing a health post as the first choice for care was one of the predictors of poor mothers' behavioral change (Table 5). Poor mothers might naturally select the health facility that is closest as their first choice; however, they also might recognize that the health post did not offer satisfactory health services. Some mothers stated during the interview that health personnel were not in the health post when they were needed. This suggests that, although the national rural health system was functioning, the services might be limited. Indeed, many developing countries still have low rates of systematic referral for child illnesses [49,50]. We should consider and re-define the roles of the rural health posts. If the rural health posts are active enough, more childhood death reductions might be accomplished [43]. Opinions on the quality of rural health posts vary. One study reported significantly lower child mortality among mothers who used a rural dispensary than those who did not [48]. Another study criticized rural health posts' restricted hours and irregular days of operation [24].

In addition, the experience of a previous child's death predicts a positive change of poorer mother's second choice. One possible reason is that mothers who have experienced a child death in the past might be more sensitive to trying to save another child's life. Determination of the severity of a childhood disease is difficult for poorer mothers [26]. Indeed, childhood disease prevention is a challenging issue for the poor. Child stunting was not identified as the predictor in our final model. Poor mothers in developing countries may not know that nutrition is a major risk factor for child disease, regardless of participation in recent discussions on child malnutrition [51,52]. Moreover, their urgent change of care-seeking might not always be successful. We observed that the number of mothers who mentioned the death of a child was much higher than the official data [24,48,53]. This is commonly shown in studies from developing countries [54,55].

### Heavy medical burden for the poor

As our data suggested, money to pay for medical care was a major complaint of the poor (Table 3). The quantified evidence in developing countries is quite limited [53,54], but our study demonstrated the burden of their out-of-pocket payments.

The median percentage of monthly medical expenditure to household income in this study (10.0%) was similar to that of a study in the republic of Georgia [55], although this is remarkably high compared to results of studies in Pakistan [56] and China [57]. Both the latter studies indicated 5% was the average proportion in relation to monthly household income. A study in Nepal [45] found that the percentage of medical expenditure to household income was 5.5%. Some of these studies suggested that the rich are more likely to pay for medical care.

Personal medical expenses reflect both health care-seeking on the demand side and the available health services on the supply side. As several existing studies have shown, those who live in poor surroundings are likely to pay more for medical treatment [45,54,55,58]. Our evidence supports this fact.

This study also may reflect that expensive drug purchases were being made and that health insurance covers only a low proportion in Nicaragua. Nevertheless, our results suggest that people in this area had high expectations of the medical care on offer (Table 3).

### Mothers' perception of medical service

Our study showed mothers' high proportion of satisfaction with medical services (Table 3, >58%). We identified different health service needs by the economic status even in this small community. Poor mothers expect easily accessible, low-cost health services, while affluent mothers expect less waiting time and high-quality health services (Table 3).

We also found a significant difference in interest in health education between the poor and rich, with the poorer mothers showing more interest than the wealthiest (Table 3, p = 0.015). A majority of mothers were interested in more health education, which may reflect the lack of current opportunities (Table 1). Importantly, health education opportunities in rural areas could mobilize poorer mothers [43]. Although it is difficult to measure the effect of health education, a recent study [26] emphasized that health education combined with regular child growth monitoring led to poor mothers' better health behaviors and better health outcomes.

Finally, as a recent study has emphasized [59], the difficulty in improving access to better health services is not a single-failure problem. Access problems are the results of multiple failures: government failure, market failure, and misunderstanding of human behaviors [59]. Changing individual behaviors is a challenging issue. Our study revealed to a certain degree this invisible, tangled yarn.

### Limitations of the study

This study has several limitations. Since our study was cross-sectional, we could not determine causality. Even though we tried to select an average area, the results cannot be fully generalized to the whole country. Our data is from 2002; however, to our knowledge, no studies that have examined poor mothers' care-seeking behavior in Nicaragua. We assigned health professionals to be the research assistants. Therefore, some mothers possibly answered questions mindful of preferable opinions for them. Still, this study covered all caretakers of children under 2 years old in the target area.

### Recommendations

As we discussed, we suggest that the government should seek to reduce the burden of medical costs for the poorest. Nicaragua still has huge economic disparities between the rich and the poor, and between rural and urban areas. The government should tackle not only fortification of the referral system but also of the social safety net to protect the human rights of poor mothers and children. To clarify the situation, and identify the priorities, further longitudinal studies are required.

## Conclusions

Determination of the severity of a childhood disease is a difficult task for mothers. The national rural health system was functioning, yet, the services may have been limited. We should consider and seek a more effective primary care system for the poor.

To encourage mothers' care-seeking behaviors in poor settings, the referral system and the social safety net need to be strengthened, and mothers need to be taught more about how to recognize the danger signs of child illness.

## Competing interests

The authors declare that they have no competing interests.

## Authors' contributions

KS carried out the research, analyzed the data, and led the writing of the article. MJ assisted with the writing. MJ and KH gave useful comments on interpretation of the data. KH managed research funds and supervised the field study. All of the authors reviewed the article and approved the final draft.

## Authors' information

KS is a Takemi Fellow (Post Doctoral Research Fellow) in Takemi Program in International Health, Harvard School of Public Health, US, and visiting researcher in Department of Community and Global Health, Graduate School of Medicine, the University of Tokyo, Japan. MJ is a Professor and Chair, Department of Community and Global Health, Graduate School of Medicine, the University of Tokyo, Japan. KH is a Senior Adviser, Human Development Department, Japan International Cooperation Agency, Tokyo, Japan.

## Author Details

**KS**: Takemi Program in International Health, Department of Global Health and Population, Harvard School of Public Health, 665 Huntington Ave. Boston, MA 02115-6021, USA.

**KS**, **MJ**: Department of Community and Global Health, Graduate School of Medicine, The University of Tokyo, 7-3-1 Hongo, Bunkyo-ku, Tokyo, 113-0033, Japan.

**KH**: Human Development Department, Japan International Cooperation Agency (JICA),

Nibancho Center Building, 5-25, Nibancho, Chiyoda-ku, Tokyo, 102-8012, Japan.

## Pre-publication history

The pre-publication history for this paper can be accessed here:

http://www.biomedcentral.com/1472-698X/10/10/prepub
